# A Single Institution Experience with a Shear-Thinning Conformable Embolic for Endovascular Embolization

**DOI:** 10.1007/s00270-025-04012-y

**Published:** 2025-03-19

**Authors:** Olivia Kola, Pratik Shukla, Humza Haque, Abhishek Kumar

**Affiliations:** 1 Rutgers - New Jersey Medical School, Newark, NJ USA; 2 Division of Vascular and Interventional Radiology, Department of Radiology, Rutgers - New Jersey Medical School, 185 South Orange Ave. MSB F-560, Newark, NJ 07103 USA; 3 Department of Radiology, Rutgers - New Jersey Medical School, Newark, NJ USA

**Keywords:** Embolization, Arterial embolization

## Abstract

**Purpose:**

To assess the safety and efficacy of Obsidio™ conformable embolic (CE) for embolization in the peripheral vasculature.

**Materials and Methods:**

A retrospective review of the first 21 patients treated with CE was performed. Eighteen (85.7%) patients were male, and median age was 61.5 years (range, 12–89 years). Technical success was defined as stasis as assessed by a static contrast column for at least 5 heartbeats on post-embolization angiography. For procedures of peripheral vascular hemorrhage, clinical success was defined as hemorrhage resolution without reintervention within 30-day follow-up.

**Results:**

Indications for embolization were peripheral arterial hemorrhage (*n* = 13), preoperative tumor embolization (*n* = 4), preoperative embolization of renal cell carcinoma prior to cryoablation (*n* = 2), redistribution of flow prior to Yttrium-90 radioembolization to prevent nontarget radiation delivery (*n* = 1), and parastomal variceal embolization (*n* = 1). Embolization was performed via 2.4 or 2.8 French microcatheters flushed with saline prior to embolization. Most procedures (20/21) utilized < 1 cc of embolic, with the quantity used ranging between 0.1 and 1.4 cc. The amount of embolic injected was determined by the embolization endpoint, i.e., filling of the vessel intended for embolization. CE was used in combination with coils placed prior to CE in 4 procedures. Follow-up was a median of 57 days (range 0–244 days). Complete stasis was achieved in 100% (*n* = 21/21) of procedures. There were no post-procedure adverse events or rebleeding.

**Conclusion:**

CE resulted in reliable vessel occlusion with no cases of rebleeding or reintervention and with no procedure-related adverse events in this series.

*Level of Evidence*: Level 4, Case Series.

**Graphical Abstract:**

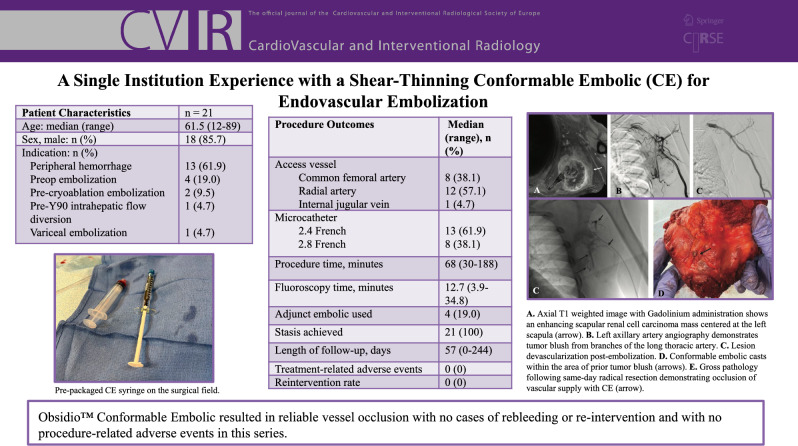

## Introduction

Injectable hydrogels exhibit shear-thinning properties, i.e., viscosity decreases under shear stress and is recovered with elimination of stress [10]. Obsidio™ conformable embolic (CE) (Boston Scientific Corp., Marlborough, MA) is a recently US Federal Drug Administration-approved shear-thinning embolic indicated for embolization of hypervascular tumors and for control of hemorrhage in peripheral vasculature. Composed of pre-hydrated bioresorbable gelatin, Laponite® nanosilicate (ECKART, Hartenstein, Germany), and tantalum powder, CE is packaged in sterile, single-use 1 cc Luer-lock syringes [8]. Preclinical in vivo animal studies show CE can withstand higher displacement pressures than coils and triggers a natural coagulation pathway leading to a concentric inflammatory reaction causing localized fibrosis of the vessel lumen [2, 3]. A recently published case series by Pal et al. details 11 patients treated with CE for peripheral hemorrhage, reporting 100% technical success and no adverse events [7]. Theirs was the first published report for the use of CE. We sought to add to the literature assessing the safety and feasibility of CE as an embolic for treatment in the peripheral vasculature.

## Materials and Methods

A single-center retrospective analysis was performed between October 2023 and February 2024 of the first 21 patients treated with CE. Embolization was performed by three interventional radiologists with 2–10 years of experience. The decision to use CE was at the discretion of the operator. Patient demographics, baseline and treatment characteristics, and clinical courses were queried from the electronic medical record, and descriptive statistics were calculated.

**Fig. 1:**
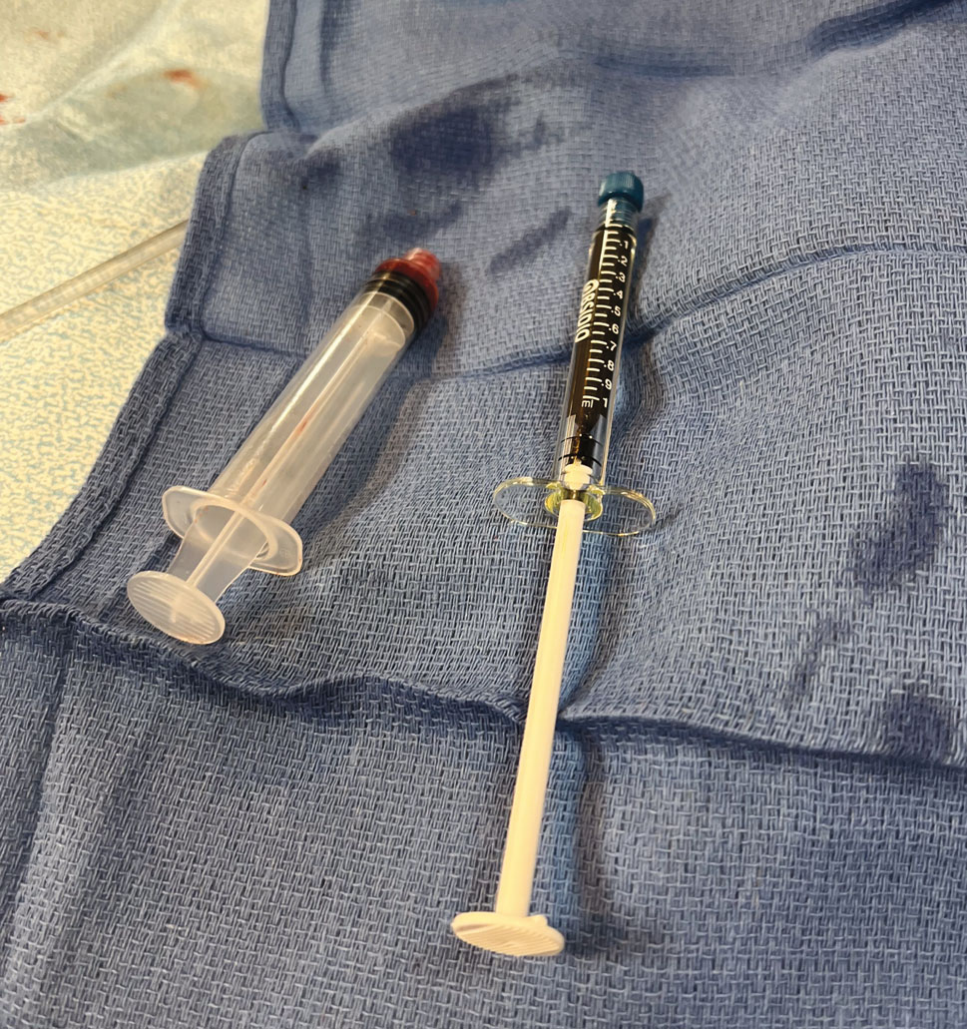
Conformable embolic syringe noted on the surgical field prior to embolization. The syringe is prepackaged and can be opened directly onto the sterile field without preparation to be attached directly to the Luer-lock hub of the injection microcatheter

A combination of 5 French base catheters with 2.4 or 2.8 French PROGREAT® (Terumo, Tokyo, Japan) microcatheters was used to deliver CE, which were flushed with saline prior to delivery. There was no preparation of either CE (e.g., perturbation) or microcatheters prior to delivery. Prepackaged 1 cc syringes of CE were attached directly to the Luer hub of the delivery catheter; CE was loaded and delivered under X-ray fluoroscopy guidance (Fig. 1). After sufficient embolic material was noted in the target vessel, the microcatheter was removed. Post-embolization angiography was performed via the base catheter. When used in combination with coils, CE was deployed following coil placement, with coil placement intended to provide a backstop to prevent distal embolization. Technical success was stasis as assessed by a static contrast column for at least 5 heartbeats on post-embolization angiography. For hemorrhage, clinical success was defined as resolution without reintervention within a 30-day follow-up period. Treatment-related adverse events were recorded through discharge and available follow-up. Adverse events were classified using criteria set forth by the Cardiovascular and Interventional Radiological Society of Europe [4]. Nontarget embolization was assessed by visually noting CE in an unintended target during the procedure or clinical sequela such as infarction on postoperative follow-up.

## Results

**Fig. 2:**
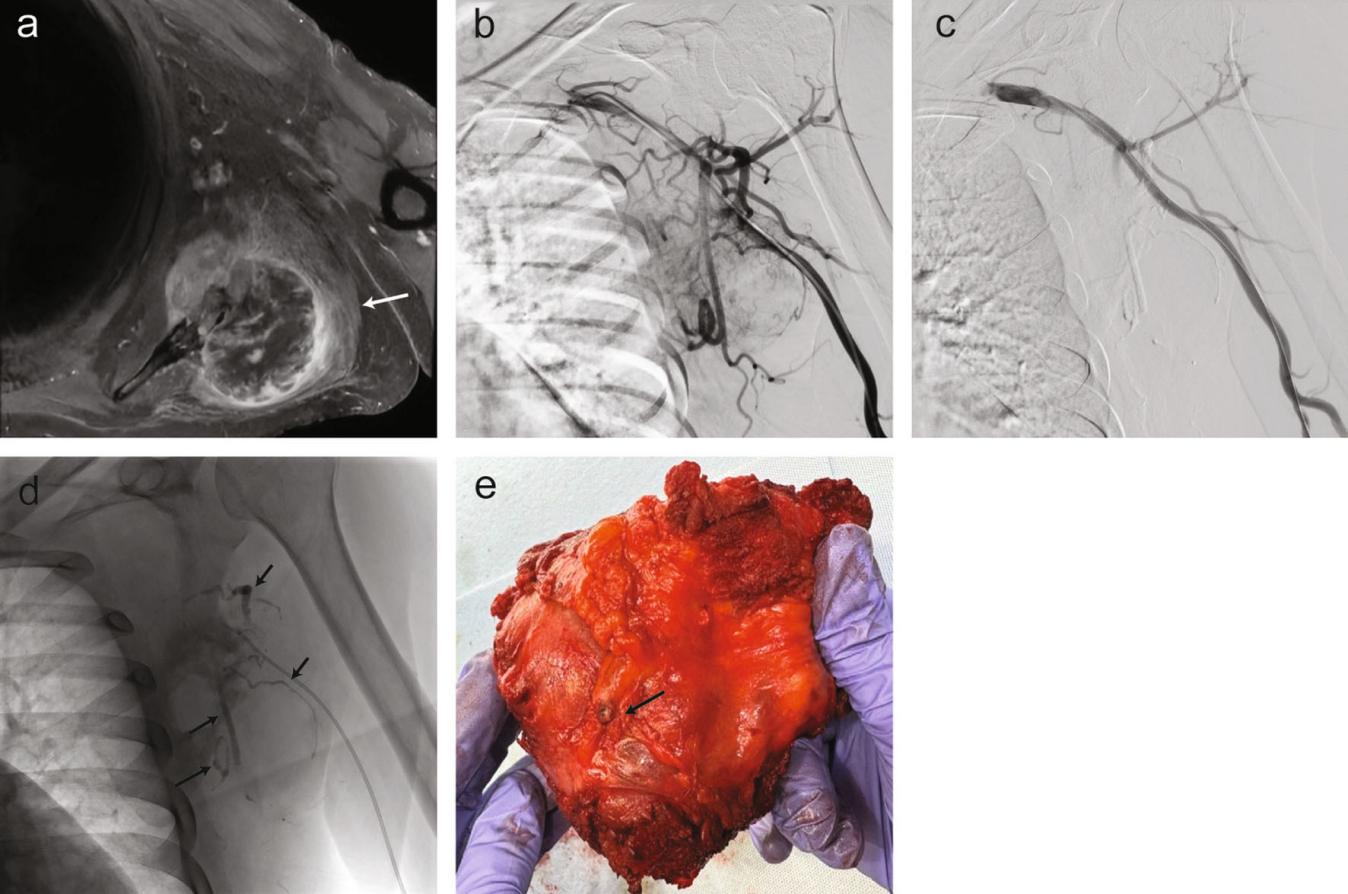
Preoperative embolization of a scapular mass in a 66-year-old male with a history of pT3aN1M1 clear cell renal cell carcinoma. (**A**) Axial T1-weighted image with gadolinium administration shows an enhancing mass centered at the left scapula (arrow). (**B**) Pre-embolization left axillary artery angiography demonstrating tumor blush originating from multiple branches of the long thoracic artery. (**C**) Post-embolization angiography via a 4 French angled catheter with a total of 1.4 cc CE delivered via 2.8 a French microcatheter demonstrating devascularization of the lesion. (**D**) Post-embolization fluoroscopic image demonstrating conformable embolic casts within the area of prior tumor blush (arrows). (**E**) Gross pathology following same-day radical resection of the metastatic renal cell carcinoma mass demonstrating occlusion of vascular supply with CE (arrow). The lesion was found to be a multilobulated tan-pink necrotic mass measuring 6.2 × 4.2 × 3.9 cm

**Fig. 3:**
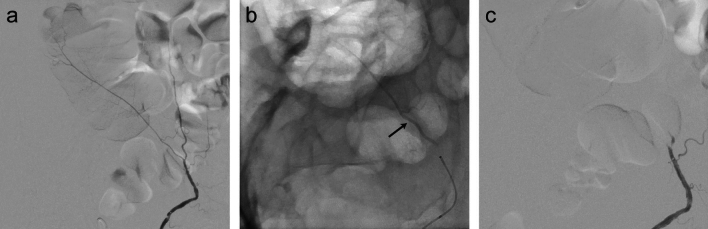
Embolization of the inferior epigastric artery for acute peripheral hemorrhage in a 57-year-old man with end-stage renal disease on hemodialysis, heart failure with reduced ejection fraction, polysubstance use, and newly diagnosed cirrhosis complicated by ascites underwent two bedside paracenteses, the second of which produced 3L of blood-tinged fluid accompanied by a drop in hemoglobin (6.0 g/dL from 7.8 g/dL). Vital signs were stable; however, computed tomography of the abdomen showed hemoperitoneum with a right rectus sheath hematoma and active arterial bleeding. (**A**) Pre-embolization anteroposterior digital subtraction angiography of the right inferior epigastric artery via a 4 French angled catheter via transfemoral access. (**B**) Fluoroscopic still image showing < 1 cc conformable embolic delivered via a 2.8 French microcatheter (arrow). (**C**) Post-embolization digital subtraction angiography demonstrating a static contrast column

**Fig. 4:**
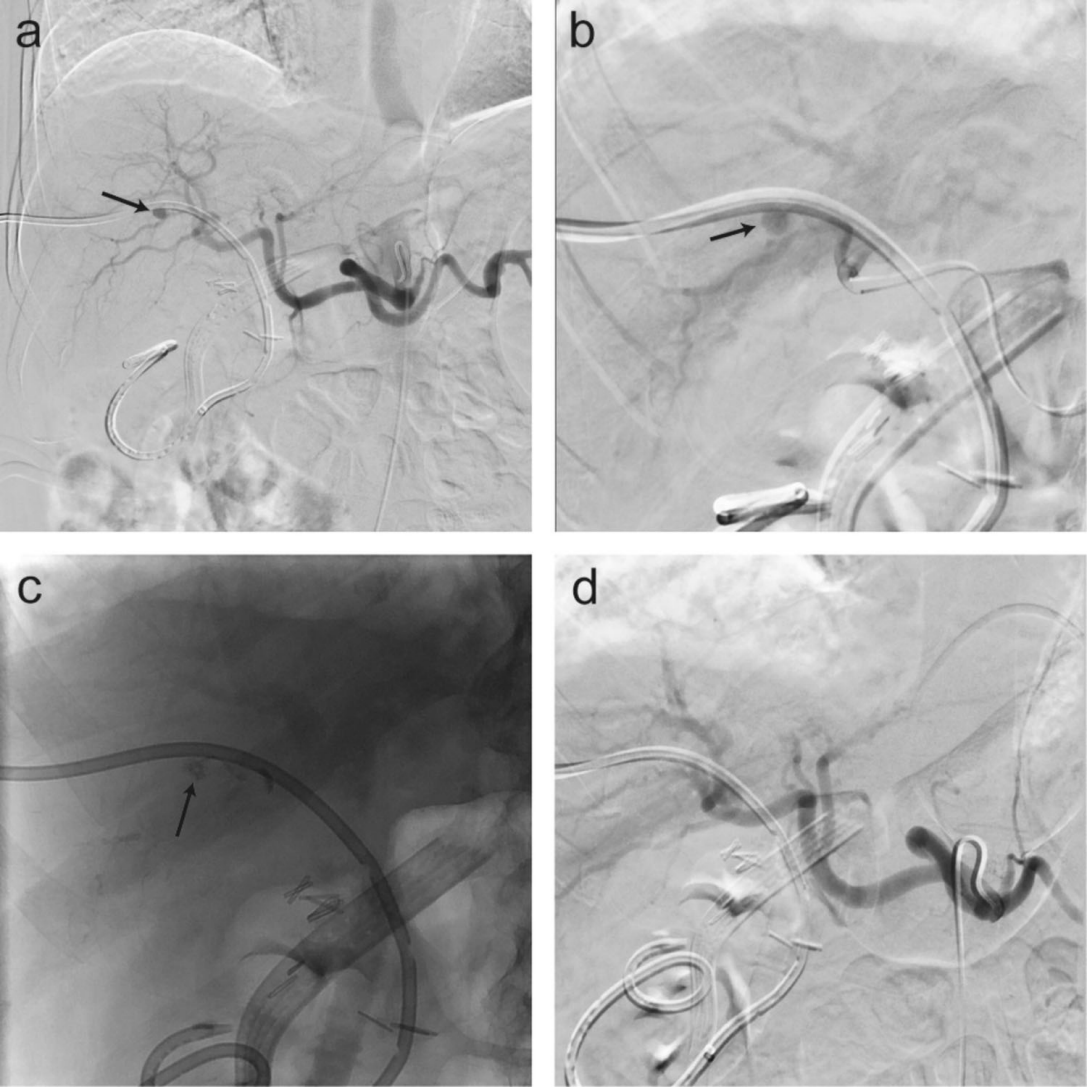
Hepatic pseudoaneurysm. (**A**) Celiac angiography demonstrates pseudoaneurysm of a right hepatic artery branch at the location of the biliary drain (arrow). (**B**) Selective angiography via 2.4 French microcatheter demonstrates a pseudoaneurysm adjacent to the drain (arrow). (**C**) Post-injection fluoroscopic still image demonstrating conformable embolic cast (arrow). (**D**) Post-embolization angiography shows successful embolization of the pseudoaneurysm

**Fig. 5:**
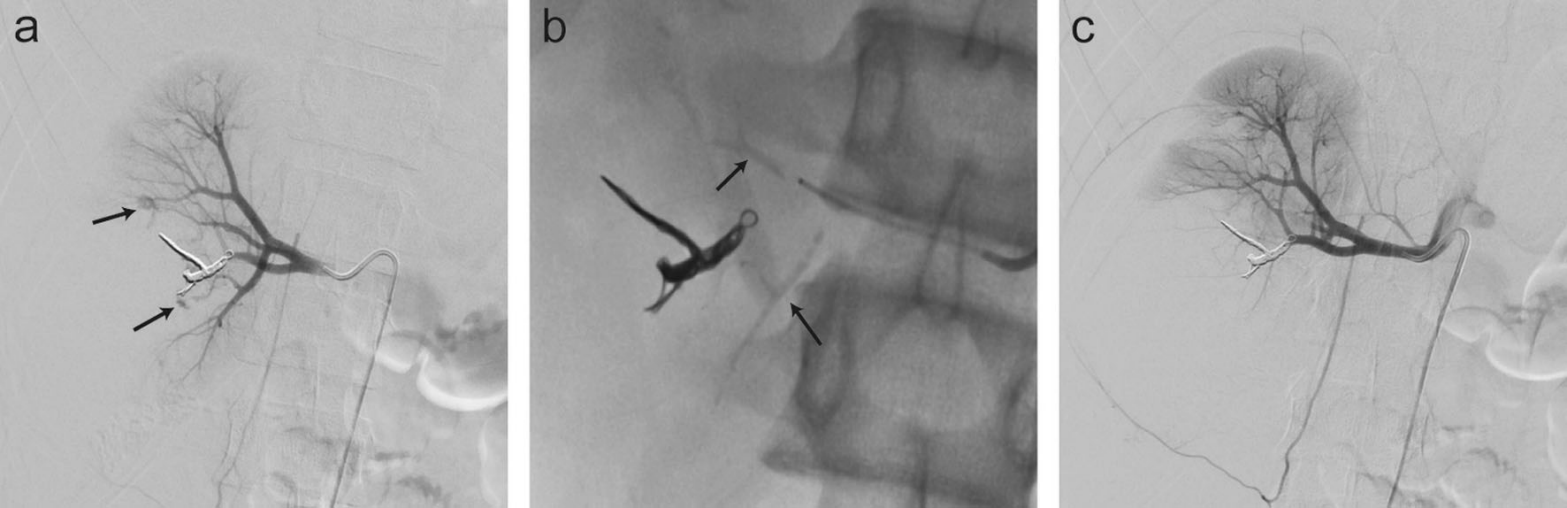
Renal pseudoaneurysms. (**A**) Renal arteriogram demonstrates pseudoaneurysms in the upper and lower pole branches (arrows). (**B**) Post-embolization fluoroscopic still image demonstrating conformable embolic cast in the upper and lower pole renal artery branches (arrows). (**C**) Post-embolization angiography shows occlusion of both embolized vessels and pseudoaneurysms

Patient demographics, procedure characteristics, and outcomes are summarized in Table 1. The volume of CE ranged from 0.1 to 1.4 cc, with one case utilizing > 1 cc. Five (23.8%) patients were coagulopathic within 24 h prior to embolization (INR > 1.5). Indication for embolization, specific vessels embolized, and adjuvant embolic for individual procedures are in Table 2. Four procedures utilized CE combined with coils (Table 2). The median follow-up period was 57 (range 0–244) days. Technical success was 100% (*n* = 21/21), and there were no treatment-related adverse events or nontarget embolization. Of the 13 patients requiring embolization for hemorrhage, 8 met criteria for clinically successful embolization, i.e., a 30-day period without hemorrhage recurrence requiring reintervention. There was not adequate length of follow-up for the 5 patients that did not meet this criterion, with follow-up for these patients ranging from 0 to 29 days; however, none of these patients required additional treatments for refractory bleeding or recanalization of target vessels. Four cases were selected to demonstrate angiographic appearance of and diverse indications for CE, including preoperative tumor embolization of a scapular mass (Fig. 2) and embolization of an inferior epigastric artery (Fig. 3), a hepatic pseudoaneurysm (Fig. 4), and renal pseudoaneurysms (Fig. 5) for peripheral hemorrhage.

**Table 1:** Patient and procedure characteristics and embolization outcomes

Patient characteristics	
Age: median (range)	61.5 (12–89)
Sex, male: n (%)	18 (85.7)
Indication: n (%)	
Hemorrhage	13 (61.9)
Preoperative embolization	4 (19.0)
Pre-cryoablation embolization	2 (9.5)
Pre-Yttrium-90 intrahepatic flow diversion	1 (4.7)
Variceal embolization	1 (4.7)
Procedure characteristics and outcomes	
Access vessel: n (%)	
Common femoral artery	8 (38.1)
Radial artery	12 (57.1)
Internal jugular vein	1 (4.7)
Microcatheter	
2.4 French: n (%)	13 (61.9)
2.8 French: n (%)	8 (38.1)
Procedure time, minutes: median (range)	68 (30–188)
Fluoroscopy time, minutes: median (range)	12.7 (3.9–34.8)
Adjunct embolic used: n (%)	4 (19.0)
Stasis achieved: n (%)	21 (100)
Length of follow-up, days: median (range)	57 (0–244)
Treatment-related adverse events: n (%)	0 (0)
Reintervention rate (%)	0 (0)

**Table 2:** Individual indications for treatment, vessels embolized, and adjuvant embolic for all procedures

Procedure	Indication	Angiographic Findings	Vessel(s) Embolized
1	Post-paracentesis hemorrhage	No active extravasation noted on angiogram. Empiric embolization for the right inferior epigastric artery was performed	Right inferior epigastric artery
2	Internal mammary artery injury secondary to cardiopulmonary resuscitation	Active extravasation from a branch of the left internal mammary artery at the level of the posterior left third rib	Proximal left internal mammary artery
3	Grade IV splenic laceration	Numerous areas of active extravasation from small perforating branches of the splenic artery	Mid-distal splenic artery^†^ *Note: Coils were placed in the mid-distal splenic artery*
4	Preoperative embolization of hypervascular scapular renal cell carcinoma metastasis (EBL 50 mL)	Tumor blush originating from multiple branches of the long thoracic artery	Distal branches (3) of the left long thoracic artery
5	Embolization prior to Yttrium-90 for prevention nontarget embolization	Accessory dorsal subsegmental branch of the segment 8 hepatic artery arising from target treatment vessel supplying the segment 5 lesion	Proximal dorsal subsegmental branch of segment 8 hepatic artery
6	Embolization of parastomal varices following TIPS	Ileocolic venous varices arising from the superior mesenteric vein	Parastomal variceal branches (2) near their origin from the superior mesenteric vein
7	Hepatic pseudoaneurysm secondary to biliary drain placement	Pseudoaneurysm of a branch off of the segment 6 hepatic artery	Origin of the pseudoaneurysm within the segment 6 hepatic artery
8	GI bleed	No active extravasation or pseudoaneurysm, hyperemia of the stomach walls. Given findings seen on endoscopy, the decision was made to empirically embolize the left main gastric artery	Left main gastric artery
9	Pelvic hematoma secondary to fall	Active extravasation from a distal branch of the right iliolumbar artery	Distal branch of the right iliolumbar artery
10	GI bleed	Active extravasation arising from the proximal superior pancreaticoduodenal artery	Proximal-mid gastroduodenal artery
11	Preoperative embolization of a multiple myeloma lesion (EBL 400 mL)	Right axillary angiography demonstrated tumor blush originating from the right anterior and posterior circumflex humeral arteries	Right proximal anterior and posterior circumflex humeral arteries
12	GI bleed	Celiac and selective gastroduodenal artery angiography did not demonstrate active arterial extravasation; however, given pre-procedure multidisciplinary discussion, the decision made to empirically embolize the right gastroepiploic and gastroduodenal arteries	CE cast at the origin of the right gastroepiploic artery extending into the mid gastroduodenal artery *Note: Coils were placed in the proximal gastroduodenal artery*
13	Grade IV renal laceration	Large traumatic arteriovenous fistula/pseudoaneurysm in the upper pole and a smaller pseudoaneurysm in the lower pole of the kidney	Mid-distal left lower pole renal artery branch
14	Preoperative embolization of metastatic lung adenocarcinoma to the left proximal femur (EBL 400 mL)	Left profunda femoris artery angiography demonstrated tumor blush with 2/3 of the tumor supplied by the lateral circumflex femoral artery and 1/3 of the tumor supplied by the medial circumflex femoral artery	Proximal medial circumflex femoral artery and mid-distal left circumflex femoral artery
15	Renal cell carcinoma embolization prior to cryoablation	Left upper pole subsegmental renal artery angiography demonstrated tumor enhancement	Entire left upper pole subsegmental renal artery branch casted
16	Renal pseudoaneurysm following GSW to RUQ	Right renal artery angiography demonstrated multiple lower pole renal artery pseudoaneurysms	Distal branches (2) of the right lower pole renal artery *Note: Coils were placed in a right lower pole renal artery segmental branch*
17	Superior bronchial artery pseudoaneurysm	Pseudoaneurysm within the right superior bronchial artery with diffuse hypertrophy and tortuosity of the bronchial arteries	Obsidio deployed from the most distally accessible point of the right superior bronchial artery, with filling of the pseudoaneurysm and vessel casting proximally
18	Splenic aneurysms w/hematoma	Splenic artery angiography demonstrated multiple aneurysms with the largest measuring up to 2.6 cm	Proximal splenic artery^†^
19	Renal laceration	Short segment dissection of the left accessory lower pole renal artery	Proximal-mid left accessory lower pole renal artery *Note: coils were placed in the distal left accessory lower pole renal artery*
20	Lower pole renal mass embolization prior to cryoablation	Right renal artery angiography demonstrated a hypervascular right lower pole renal mass	CE injected into right lower pole segmental and multiple subsegmental renal artery branches
21	Embolization prior to open biopsy of a metastatic pelvic mass (EBL 5 mL)	Right internal iliac artery providing dominant supply to the right pelvic mass via proximal branches of the right superior gluteal artery	Right superior gluteal artery branches (2)

Procedures appear in chronological order. Numbers given in parentheses indicate conformable embolic was delivered to each branch individually. All coils were placed prior to conformable embolic. Operative estimated blood loss is noted for patients that underwent preoperative embolization. GI = Gastrointestinal, TIPS = transjugular intrahepatic portosystemic shunt, GSW = gunshot wound, RUQ = right upper quadrant, EBL = estimated blood loss

^†^ Vessel diameter > 3 mm

Of those who underwent preoperative embolization, intraoperative estimated blood loss ranged from 50 to 400 cc with no operative complications noted (Table 2). A patient who underwent intrahepatic flow diversion had successful radioembolization two weeks post-treatment with no radiotracer activity in nontarget areas. In two patients who underwent cryoablation following pre-procedure embolization, no hemorrhage was noted on post-procedure CT imaging. A patient who underwent parastomal variceal embolization had no further bleeding in the follow-up period (198 days).

## Discussion

Our study results support previously published data on CE use in peripheral vasculature. Pal et al. recently reported CE for embolization of hemorrhage in 11 patients with no adverse events and 100% success [7]. Similarly, in our study, CE embolization was clinically successful in 13/13 patients with peripheral vasculature hemorrhage. In addition, we reported use of CE for indications beyond active hemorrhage in the vasculature across various vessel diameters. No treatment-related adverse events occurred, including hypersensitivity, reflux, nontarget embolization, embolic migration, catheter entrapment, or toxicity.

CE is prepackaged in Luer-lock syringes and deployable using standard microcatheters, minimizing preparatory delay. Onyx™ and LAVA require dimethyl sulfoxide-compatible microcatheters and syringes. When deploying glue, catheters must be thoroughly flushed with dextrose solution to avoid premature polymerization and subsequent catheter occlusion or trapping. Another advantage is the lack of streak artifact on CT, which is a significant drawback of coils and liquid embolics [5]. However, unlike Onyx and LAVA, it is not possible to alter the viscosity of CE to adjust the degree of vascular penetration, potentially limiting its use for more distal embolization. The current recommendation is for usage in vessels ≤ 3 mm in diameter, limiting its versatility. With utilization of coils as backstop, CE may serve as an adjunct material for embolization in higher flow vessels.

The force of injection and viscosity of CE are different than liquid agents; embolization relies on conformation to the vessel lumen rather than interaction with blood. Although off-label, both Onyx and glue have been described as safe and feasible for embolization in peripheral vasculature; however, glue carries a risk for catheter adherence or occlusion, whereas CE is nonadhesive, offering a potential advantage [6]. Ethylene vinyl-alcohol copolymers (e.g., Onyx, PHIL) have minimal risk of microcatheter occlusion or trapping, but reflux of Onyx is poorly controlled and can lead to nontarget embolization [9]. Although there were no instances of nontarget embolization in this cohort or the previously published case series. However, it should be noted CE was recently recalled for use in lower GI bleeds, specifically when administered by the aliquot technique, as there is report of increased risk nontarget embolization resulting in bowel ischemia [1, 7]. The aliquot technique involves filling part of the dead space of the microcatheter and pushing embolic forward with saline. The shear stress forces between the saline and CE may create a diluted material, allowing for more distal, and therefore nontarget, embolization [7]. The standard injection technique involves connecting the CE syringe to the microcatheter and delivering with steady force until the desired vessel fill. The microcatheter is then removed, and a confirmation angiogram is performed via the base catheter.

This study is limited by its retrospective design, single-center setting, lack of controls, small size, and short follow-up period. Clinical success was difficult to define and standardize for procedures apart from acute hemorrhage embolization. Enrollment in the prospective, multicenter CE Conformable Embolic Registry (OCCLUDE) is underway and likely to further elucidate the utility and safety profile of CE.

## Conclusion

CE is a feasible embolic option with reliable vessel occlusion that appears to be safe and effective.
